# Repression of HIV-1 reactivation mediated by CRISPR/dCas9-KRAB in lymphoid and myeloid cell models

**DOI:** 10.1186/s12977-022-00600-9

**Published:** 2022-06-22

**Authors:** Lendel Correia da Costa, Larissa Maciel Bomfim, Uilla Victoria Torres Dittz, Camila de Almeida Velozo, Rodrigo Delvecchio da Cunha, Amilcar Tanuri

**Affiliations:** Departamento de Genética, Laboratório de Virologia Molecular, Instituto de Biologia, Universidade Federal do Rio de Janeiro (UFRJ), Av Carlos Chagas Filho 373, CCS, Bloco A, Sala 121, Ilha do Fundão, Rio de Janeiro, RJ 21941-902 Brazil

**Keywords:** HIV-1, Block and lock, Latency, LRA, CRISPR, KRAB, Repression

## Abstract

**Background:**

Despite antiretroviral treatment efficacy, it does not lead to the complete eradication of HIV infection. Consequently, reactivation of the virus from latently infected cell reservoirs is a major challenge toward cure efforts. Two strategies targeting viral latency are currently under investigation: the “shock and kill” and the “block and lock.” The “Block and Lock” methodology aims to control HIV-1 latency reactivation, promoting a functional cure. We utilized the CRISPR/dCas9-KRAB platform, which was initially developed to suppress cellular genes transcription, to block drug-induced HIV-1 reactivation in latently infected T cells and myeloid cells.

**Results:**

We identified a set of five sgRNAs targeting the HIV-1 proviral genome (LTR1-LTR5), having the lowest nominated off-target activity, and transduced them into the latently infected lymphoid (J-Lat 10.6) and myeloid (U1) cell lines. One of the sgRNAs (LTR5), which binds specifically in the HIV-1 LTR NFκB binding site, was able to promote robust repression of HIV-1 reactivation in latently infected T cells stimulated with Phorbol 12-Myristate 13-Acetate (PMA) and Ingenol B (IngB), both potent protein kinase C (PKC) stimulators. Reactivation with HDAC inhibitors, such as SAHA and Panobinostat, showed the same strong inhibition of reactivation. Additionally, we observed a hundred times reduction of HIV-1 RNA expression levels in the latently infected myeloid cell line, U1 induced with IngB.

**Conclusion:**

Taken together, our results show that the KRAB fused CRISPR/dCas9 system can robustly prevent the HIV-1 latency reactivation process, mediated by PMA or IngB and SAHA or Panobinostat, both in myeloid and lymphoid HIV-1 latently infected cells. In addition, we demonstrated that KRAB repressor protein is crucial to reactivation resistance phenotype, and we have identified some useful hotspots sequences in HIV-1 LTR for the design sgRNAs.

**Supplementary Information:**

The online version contains supplementary material available at 10.1186/s12977-022-00600-9.

## Background

Viral reservoirs and HIV-latency are the major challenges towards a cure. HIV reservoirs are established when active CD4 + T cells are infected and turn into a resting state as a memory CD4 + T cells, harboring a capable, but non-replicative, HIV-1 provirus [1, 2].

Viral latency can be established before or after the provirus integration [3] when a stable and replicative HIV-1 provirus is blocked at transcriptional or/and translational levels. Blocking HIV-1 transcription is the most frequent mechanism to induce viral latency and can happen in several ways. The viral protein Tat interacts with the trans-activation response element (TAR) in HIV-1 mRNA, increasing RNA polymerase II (RNApol II) processivity and transcription elongation, hence low concentrations of Tat induce HIV-1 latency [4]. Besides, modulation of host transcription factors (TF) required for HIV-1 gene expression, such as Nuclear factor-*kappa* B (NF-kB) and nuclear factor of activated T cell (NFAT), can also induce HIV-1 latency [5, 6]. These transcription factors are regulated by inhibitors or post-translational modifications in resting T cells [7, 8]. HIV-1 transcription can also be reduced by repressive TF, such as yin yang 1 (YY1), late SV40 factor (LSF and C-promoter binding factor (CBF) [9, 10], that recruit histone deacetylases (HDACs) to HIV-1 long terminal repeat (LTR) promotor, limiting RNApol II access [11]. Additionally, post-translational modification of histones that impact the interaction of nucleosomes with DNA makes HIV-1 LTR more or less accessible to TF and cellular transcription machinery and can directly impact latency establishment [12].

Other molecular processes that impact HIV-1 transcription possibly influence its latency, such as distal transcription elongation, multiple splicing, euchromatin and heterochromatin modulation and HIV-1 provirus integration site in the cellular genome [13, 14].

HIV-1 latency is the main barrier preventing a cure for HIV infection, and there are two main strategies to overcome this issue. The “Shock and kill” aims the eradication of latent reservoirs by the administration of latency reversing agents (LRA). ART administration prevents de novo infections and purges cells harboring the reactivated virus via active viral replication and indirectly via the host immune system. In contrast, the “block and lock” strategy is based on the impairment of some HIV-1 transcription factors in order to promote a permanent latent state, even in the presence of LRA and absence of ART. Despite the success of both models in primary cells, clinical applications must be further evaluated [13, 15, 16].

CRISPR-Cas9 technology uses short sequences of 20 bases called single guide RNA (sgRNA) and was first used as an engineered molecular scissor for gene disruption to promote protein knockout [17]. This molecular tool has been adapted and now can be used not only to generate knockouts but also to modulate transcription activation and repression, by utilizing a deactivated *Streptococcus pyogenes* Cas9 (dSpCas9) fused with a repressor or an activator protein [18].

In the context of CRISPR/dSpCas9 repressive transcription tools, the most frequent repressor is the kruppel associated box (KRAB) domain, which naturally occurs in association with zinc finger proteins (ZFP), functioning as a transcription repression factor. KRAB plays a crucial role inducing heterochromatin state of the proximal DNA sequence by recruiting KRAB associated protein 1 (KAP1), also known as TRIM28. This protein interacts with other repressive complexes such as heterochromatin protein 1 (HP1) and histone methyltransferases (HMT), which promotes the amplification of trimethylated H3K9 marker and chromatin remodeling with maximum inhibition effect ranging from − 50 to − 100 bp region downstream of transcription start site (TSS) [19]. Altogether, these tools based on DNA specific recognition and repressive epigenetic markers induction could act as a deep latency inducing strategy [20, 21]. Hence, the present study aims to provide a possible use of CRISPR/dCas9 DNA recognition system fused with KRAB domain to maintain a repressive state in a latent-infected lymphoid and myeloid cell models, upon PKC agonists or HDAC inhibitor (HDACi) stimulation.

## Methods

### Materials and reagents

The cell line HEK293T used in the present study was obtained from ATCC cell bank and maintained in DMEM medium (Dulbelcco’s Modified Eagle Medium, 11995073, ThermoFisher, MA, USA) supplemented with 10% of fetal bovine serum (A31608, ThermoFisher, MA, USA). J.Lats 10.6, a lymphocyte cell lineage, and the myeloid strain U1 cells, (derived from U937 myeloid cells chronically infected with an HIV-1 clone) used in the study were kindly provided by Dr. Lucio Gama (Johns Hopking Medical School, MD, USA) and originally obtained from the AIDS reagent program of the National Health Institute of the United States of America (NIH, MD, USA). These cells were maintained in RPMI 1640 medium supplemented with 10% fetal bovine serum. Unless stated otherwise, all cell lines were cultured without antibiotics and kept in a conventional cell culture incubator at 37 °C and 5% CO_2_ atmosphere. The cells were routinely tested for mycoplasma contamination using MycoAlert^®^ Mycoplasma detection kit (LT07-318, Lonza, FL, USA).

The phorbol esters used to induce the activation of HIV-1 promoter in latent cells were Ingenol B (IngB) (Kyolab—BR) at 1 μM and phorbol 12-myristate 13-acetate (PMA) (P8139, Merck, MO, USA) at 1 μg/μL. Suberoylanilide hydroxamic acid (SAHA—SML0061, Merck, MO, USA) and Panobinosat (Pano) (SML3060, Merck, MO, USA) were used at concentration of 5 μM and 0.15 μM, respectively [22].

The CRISPR vector, pLV hU6-sgRNA hUBC-dSpCas9KRAB-T2a-Puro named here as pdSpCas9Krab (pLV_hU6-sgRNA_hUbC-dSpCas9-KRAB-T2A-PuroR was kindly provived by Dr. Charles Gersbach (Addgene plasmid #71236; http://n2t.net/addgene:71236; RRID: Addgene_71236). In addition, pVSV-G and psPAXv2 plasmids were also acquired through Addgene platform. It is important to note that the Cas9 cloned in the vector was from *Streptococcus pyogenes* as referenced in a previous paper [23].

### sgRNAs and AAVS1 locus selection

To identify and design sgRNAs that would be complementary to the HIV-1 genome, the HIV-1 subtype B sequence, clone HXB2, (accession number AF033819 Los Alamos database) was submitted to the CRISPR Pick tool, available in the Broad Institute genetic disorder platform (GPP). This platform designs and ranks the possible sgRNAs minimizing off targets in human genome by aligning the predicted sgRNAs against the human genome sequence (GRCh38—NCBI RefSeq v.10920210514). The same platform was used to design a negative control sgRNA for adenovirus-associated integration site 1 (AAVS1) locus, commonly used as negative control in double strand brake induced by CRISPR/Cas9 system [24].

This region in human genome is inserted in protein phosphatase 1 regulatory subunit 12C (PPP1R12C) intron 1 and is described as a “safe harbor” which means that any changes in the DNA’s structure in this region will not impact the cell.

### Assembly of sgRNAs in the pdSpCas9Krab expression vector

**Fig. 1:**
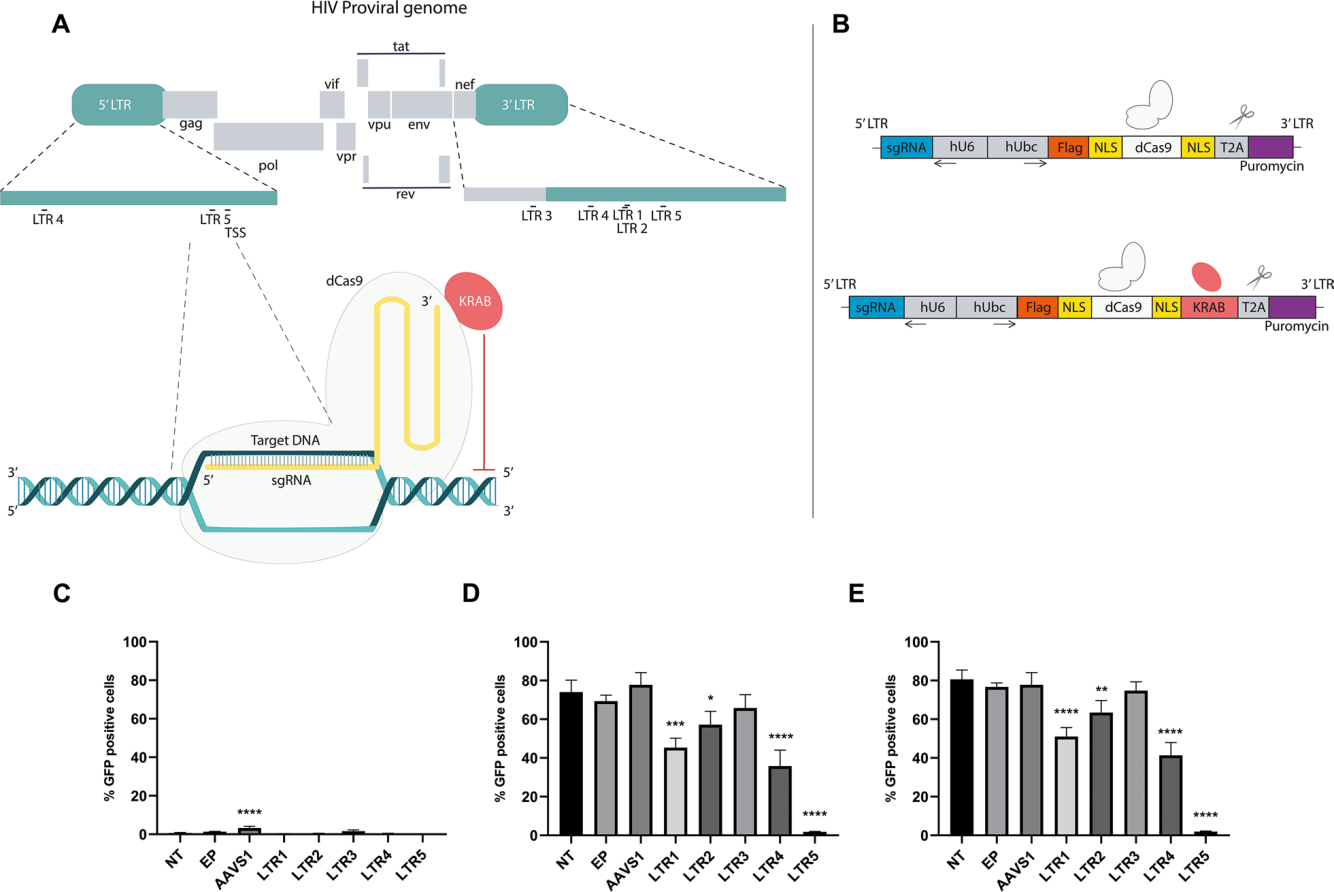
CRISPR sgRNAs constructs repress GFP expression in J.Lat 10.6 cells. **A** A schematic representation of HIV-1 provirus genome (HXB2) indicating the five predicted sgRNA ligation sites and a illustrative scheme of the used CRISPR system: CRISPR sgRNA, dCas9 and KRAB domain **B** Schematic representation of vector construct (pdSpCas9KRAB and pdSpCas9Ko/KRAB) **C** GFP background expression of each previous sgRNA (LTR1-LTR5) transduced untreated control J.Lat 10.6 cells. **D** GFP expression of each sgRNA (LTR1-LTR5) transduced J.Lat 10.6 cells treated with 1 µM PMA. **E** GFP expression of each sgRNA (LTR1-LTR5) transduced J.Lat 10.6 cells treated with 1 µM IngB. The statistical analysis was done using one-way ANOVA comparing mean of NT with AAVS1 and LTR1 to LTR5 (*p < 0.03; **p < 0.0053; ***p < 0.0003; ****p < 0.0001). The mean values of two technical measures of three independent biological experiments are shown

The pdSpCas9Krab vector expresses the fused dCas9 of *Streptococcus pyogenes* and KRAB domain, both being regulated by the UbC promoter. This vector also expresses a self-cleaving T2A catalytic and a puromycin resistant gene in the same open reading frame (ORF) of dSpCas9 and KRAB. The sgRNA is regulated by the U6 promoter that occurs in the same vector, but in the opposite sense of dSpCas9 ORF (Fig. 1B). To insert the nucleotide sequence for the sgRNA of choice, 1.5 μg of the vector pdCas9KRAB was digested with 10 UI/μL of BsmBI (R0739S, New England Biolabs, MA, USA) following the manufacture’s procedures. The sgRNA protospacer were synthetized as independent oligos by Integrated DNA technologies (USA). After the digestion, lyophilized sgRNAs oligos (Additional file 1) were resuspended in H_2_O DEPC to make 100 μM stocks. T4 PNK (M0201S, New England Biolabs, MA, USA) was used for the phosphorylation step following to manufacturer’s instructions. Then, the reaction was submitted to 95 °C for 5 min and 5 °C for 1 min to join the two DNA strands. The ligation of the sgRNAs sequences to the digested expression vector was performed using T4 DNA Ligase enzyme (M0202S, New England Biolabs, MA, USA), following manufacturer’s procedures. Chemically competent bacteria JM109-prepared using Mix & Go Competent Cells—JM109 strain kit (T3003, Zymo Research, CA, USA)—were transformed with 2 μL of ligation product using manufacturer’s recommendations. Positive bacteria were selected by plating in LB + 50 μg/ml ampicillin plates kept at 37 °C for 24 h and confirmed by colony PCR followed by electrophoresis on a 2% agarose gel, where fragments of approximately 280 bp were expected (data not shown). These confirmation PCR reactions used the U6 forward primer and the one of sgRNA strand as a reverse primer [LTR (1–5) Reverse].

### KRAB sequence excision

The sequence of the repressor protein KRAB was removed from pdSpCas9KRAB using 10 UI of NheI (Promega, WI, USA) and the resulting plasmid was relinked with 1 UI of T4 ligase (M1794, PROMEGA, WI, USA), generating the vector pdSpCas9koKRAB. The excision was confirmed by sequencing the plasmid using the BigDye Terminator protocol (ThermoScientific, MA, USA), according to the manufacturer’s recommendations (data not shown).

### Lentiviral vectors production

HEK293T cells were seeded at 8.0 × 10^5^ cells/mL per well in 6-well plates, 24 h before transfection using calcium chloride protocol. The LTR1-5sgRNA/dSpCas9KRAB expression vectors were incubated with a mix solution containing 9 μL of 2.5 M CaCl2, 8 μg of total plasmid (3.53 μg of each expression vectors, 1.76 μg of VSV-G and 2.7 μg of psPAX) and H_2_O DEPC to a final volume of 90 μL. The same volume of 2X Hepes Buffer Saline (HBS) was added for DNA precipitation. The mixture was incubated at room temperature for 15 min and dripped gently into the well, which had the culture medium previously replaced by a 1 mL of Opti-MEM (31985070, ThermoFisher, MA, USA). The transfection solution was kept in contact with cells for 6 h and after this time replaced by 2 ml of complete medium. After two days, the supernatant from each well was collected, filtered with a 0.22 μm filter and the solution containing the lentiviruses was frozen at − 80 ºC until further use.

### Transduction of lentiviral vectors in lymphoid and myeloid cells

The cells used for virus transduction with sgRNA/pdSpCas9KRAB construct were J.Lat 10.6 and U1. For this purpose, 2.5 × 10^5^ cells were plated in a 12-well plate in a final volume of 1 mL. Then, 8 μg/mL polybrene (Merck—H9868) and 500 μL of the lentivirus solution was added to the culture medium. The virus and cell solution were then submitted to centrifugation at 1000*g* for 2 h at room temperature. After 24 h, 0.5 μg/mL of puromycin (P8833, Merck, MO, USA) was added to select transduced cells. The selection of stable transduced cells took approximately two weeks and was confirmed by PCR when 100 bp fragments should be amplified from selected cells. The forward primers were one sgRNA strand and the U6 promoter reverse primer (Additional file 3: Fig. S1).

### Measurement of GFP from induced J-Lat 10.6 cells

Non-transduced (NT)—wild type J.Lat 10.6 cells or J.Lat 10.6 cells transduced with empty vector (EP), empty vector knockout KRAB (EPko/KRAB), AAVS1 sgRNA or LTRs sgRNA with and without KRAB cells (LTR or LTRko/KRAB) were plated at a 10^5^ cells/mL, in 24-well plates and separated into three experimental groups: the untreated group, that did not receive the reactivating drug (negative control); the PMA group treated with 1 μg/μL of PMA and the IngB group which received 1 μM of ingenol B. After 24 h cells were centrifuged at 300*g* for 4 min and resuspended in 100 μL of PBS. Reactivation was quantified by GFP fluorescence intensity using a BD Accuri C6 flow cytometer (BD Biosciences, NJ, USA).

### HIV-1 RNA detection in U1 cell line model

The U1 cell activation process was conducted as previously described by Abreu et al. [25]. U1 NT cells and U1 LTRs were plated, in duplicate, at a concentration of 2.0 × 10^5^ per well. Two conditions per cell type were adopted: without treatment or with 1 µM IngB for 48 h. After treatment cells supernatant were frozen at − 80 °C. Before the RTqPCR test, we diluted the supernatant samples by 1000 times. HIV-1 viral load was estimated by HIV-1 Real Time Amplification Kit through m2000 Real Time System (Abbott, IL, USA). The primers target was manufactured by Abbott to the highly conserved region of *pol integrase* gene region. Primers sequences were not provided.

### Statistical analysis

Statistical calculations were performed using one-way or two-tailored unpaired Student T test using Graph Pad Prism version 8.0.0. *p* values ≤ 0.05 were considered statistically significant. Experiments were performed in three independents biological replicates with two technical replicates per samples. Statistical data analysis of untreated J.Lat 10.6 ko/KRAB cells experiments were performed in two independent biological replicates with two replicates per samples.

## Results

### Design and selection of sgRNAs binding to HIV-1 LTR

In order to find the best sgRNAs that bind to HIV-1 provirus genome with maximum on target and minimum off target human genome ligation, we performed an in silico screening using the GPP (Genetic Perturbation Platform) sgRNA designer tool from Broad Institute. Through in silico analysis of the files generated by the platform, 98 possible sgRNAs were obtained (Additional file 2). First, we performed a screen to analyse only the sgRNA which bind in HIV-1 LTR or near locations. More off-targets mean a lower score assigned by the program and a lower placement of this sgRNA, so among these 98 possibilities, the five best ranked sgRNAs were chosen based on the major parameters established above and were named LTR1 to LTR5 (Table 1). LTR1 and LTR2 sgRNAs bind to the U3 region of the 3′ LTR, (+ 8924 bp and + 8921 bp upstream of HIV-1 RNA TSS, respectively). LTRs 3 binds to the region encoding NEF viral protein,  + 8525 bp, LTR4 binds to the U3 region of the 5′LTR (− 391 bp) and 3′LTR (+ 8695 bp) and finally, LTR5 sgRNA binds to NF-kB transcription factor binding sites in the 5′LTR (− 92 bp) and 3′LTR U3R region (+ 8993 bp). (Fig. 1A; Table 1).

**Table 1:** Constructs sequences, ligation sites and position to HIV-1 TSS

Vector name	sgRNA sequence	HIV-1 provirus ligation site^a^	HIV-1 genome position	Position from 5′LTR TSS
pLTR1	5′CCACGTGATGAAATGCTAGG3′	3′LTR U3	9360–9379	+ 8924
pLTR2	5′CCGCCTAGCATTTCATCACG3′	3′LTR U3	9357–9376	+ 8921
pLTR3	5′TGCCTGGCTAGAAGCACAAG3′	NEF	8961–8980	+ 8525
pLTR4	5′CTGTGGATCTACCACACACA3′	5′LTR U3/3′LTR U3	45–64/9130–9149	− 391
pLTR5	5′CTACAAGGGACTTTCCGCTG3′	5′LTR U3R 3′LTR U3R	344–363/9429–9448	− 92

pLTR—expression vector containning sgRNA, the dCas9 and KRAB domain

^a^Sequence from subtype B group M HIV-1

### HIV-1 latency can be maintained by specific CRISPR action in a lymphoid model

To understand whether the sgRNA inserted in the repression vector could limit HIV-1 replication, J.Lat 10.6 cells were transduced with the repressor constructs LTRs 1 to 5 and were then subjected to PMA and IngB reactivation. Reactivation was not induced by solely the presence of the constructs alone and absence of induction as seen in Fig. 1B, despite it was observed a slightly reactivation, 4% of GFP on average, in untreated AAVS1 clone. Although this result was statistically significant, it was considered as background of reactivation from the J.Lat 10.6, since when reactivated with PMA or IngB, there were not any difference in reactivation between NT, EP or AAVS1 clones. After induction with either PMA or Ing, LTR3 cells were still activated and no statistically significant difference in GFP expression was detected when compared to treated NT and AAVS1 controls (Fig. 1C and D). Neverthless, in LTR1, LTR2, LTR4 cells treated with LRA drugs, a statistically significant reduction in GFP expression was seen (p < 0.05) compared to the treated NT (28.70%, 16.87% and 38.27%, respectively). Interestingly, we obtained an even better inhibition in LTR5 cells, where decreases of 72.3% and 78.7% in GFP expressing cells were detected after PMA and IngB treatments, respectively (Fig. 1C and D; p < 0.0001). No reduction in fluorescence was detected in cells transduced either with empty vector or AAVS1 sgRNA treated with PMA and IngB compared to the non-transduced cells, as expected.

### KRAB domain impact in the maintenance of HIV-1 latency

**Fig. 2:**
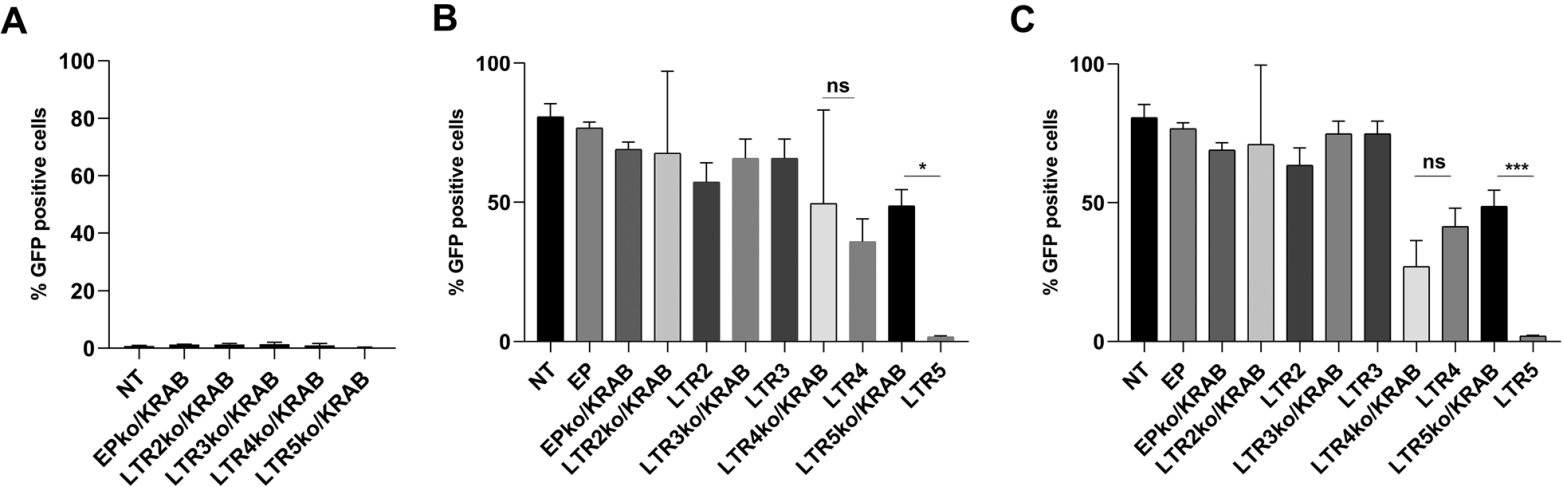
KRAB depletion abolishes CRISPR/dCas9 system repression. **A** GFP expression of each sgRNA (LTR2-LTR5) transduced J.Lat 10.6 cells treated with 1 µM PMA. pdCas9ko/KRAB is the sgRNA and dCas9 expression vector without the KRAB domain sequence and was used as a negative control **B** GFP expression of LTR2 to LTR5 J.Lat 10.6 cell clones with and without KRAB domain expression upon PMA stimulation. **C** GFP expression of LTR2 to LTR5 J.Lat 10.6 cell clones with and without KRAB domain expression upon IngB stimulation. The statistical analysis of **A**–**C** was performed using one-way ANOVA comparing mean of each column with mean of each other column with LTR2ko/KRAB to LTR5ko/KRAB for PMA group (**p < 0.0004; ****p < 0.0001) and for IngB group (**p < 0.0058; ***p < 0.0007; ****p < 0.0001). The statistical analysis comparing LTR4 (ns—PMA and IngB) or LTR5 (*p < 0.0302—PMA/*** *p* < 0.0004—IngB) with and without KRAB was performed by one-way ANOVA with multiple comparisons). The mean values of two technical measures of three independent biological experiments are shown

To confirm that the suppression of GFP expression in induced J.Lat 10.6 cells was a direct result of the presence of the CRISPR constructs we removed KRAB repressor sequence from the LTR2, LTR3, LTR4, LTR5 vectors, generating sgRNA knockouts KRAB (LTR2-LTR5ko/KRAB). Cells transduced with these vectors were treated with PMA (Fig. 2B) or IngB (Fig. 2C). No significant differences were found for all the constructs without KRAB domain comparing against NT after PMA induction of reactivation and approximately the same % of GFP positive cells were observed (Fig. 2B). In both LTR4ko/KRAB and LTR5ko/KRAB, we detected a statistically significant GFP repression in reactivation in the absence of KRAB domain comparing against NT, in IngB treatment, 27% and 48.8%, respectively (Fig. 2C). To improve the analysis, whether the KRAB was relevant or not to the repression, we combined the data from Fig. 1C and D with the % of GFP positive cells of construct without KRAB domains stimulated with PMA or IngB, respectively. Upon PMA and IngB stimuli (Fig. 2B), we could not observe a statistically significant GFP reduction in LTR4ko/KRAB comparing with LTR4 (*ns*), otherwise we noted a GFP reduction between LTR5ko/KRAB and LTR5 (*p* < 0.03021/*p* < 0.0004). These results might suggest that the presence of KRAB domain was central for LTR5 sgRNA inducing repression but not for LTR4which only the dSpCas9 presence could promote some inhibition.

### CRISPR sgRNA LTR5 repressor prevents HIV-1 reactivation by non PKC agonists LRAs

**Fig. 3:**
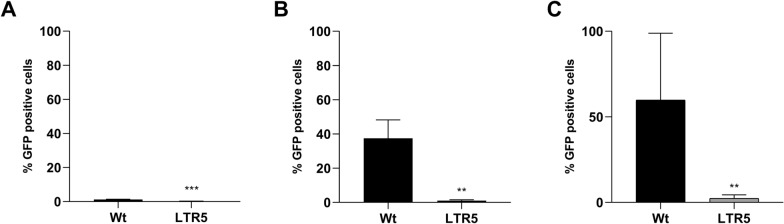
CRISPR LTR5 J.Lat 10.6 cells still promotes HIV-1 latency repression in the presence of HDAC inhibitors. **A** GFP expression of each sgRNA LTR5 transduced J.Lat 10.6 cells untreated. **B** GFP expression of sgRNA LTR5 transduced J.Lat 10.6 cells treated with 5 µM SAHA for 24 h. **C** GFP expression of sgRNA LTR5 transduced J.Lat 10.6 cells treated with 0.15 µM Panobinostat 24 h. The statistical analysis was done using Student-T test comparing mean LTR5 with NT (**p < 0.05; ***p < 0.0008). The mean values of two technical measures of three independent biological experiments are shown

After confirmation of which was the most effective sgRNA repressing HIV-1 reactivation, we investigated whether this repression could be observed by stimulation of LRAs other than PKC agonists. LTR5 J.Lat 10.6 cells were treated with SAHA and Panobinostat (Pano), both potent HDAC inhibitors. We found that in LTR5 J.Lat 10.6 cells reactivation mediated by SAHA and Pano was reduced 35 and 24 times, respectively when compared with NT cells, measured as % of GFP positive cells (Fig. 3B and C). These results show that the CRISPR Cas9-KRAB system can suppress HIV-1 latency reactivation by different classes of LRAs.

### HIV-1 latency can be maintained by specific CRISPR action in a myeloid cell model

**Fig. 4:**
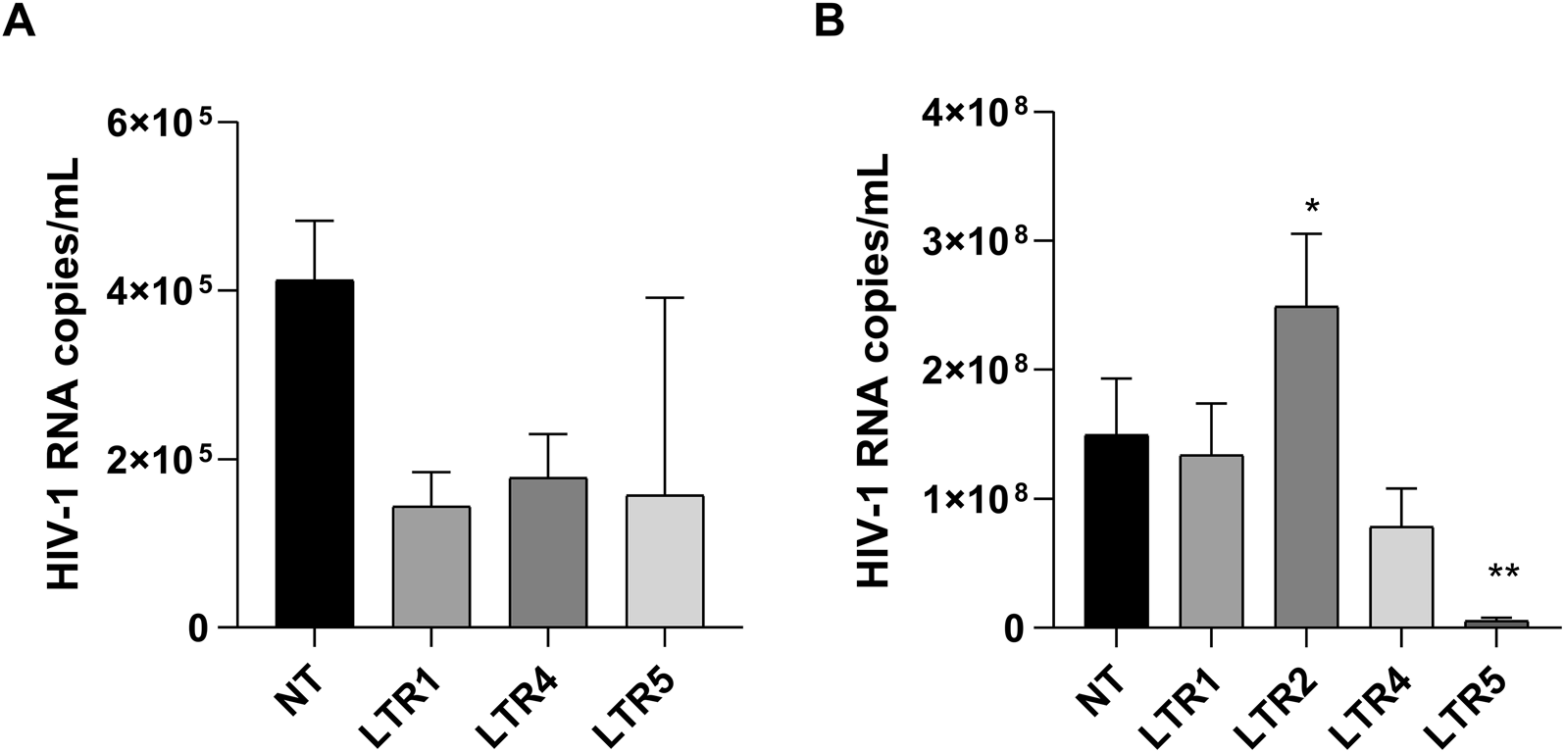
CRISPR sgRNAs repress HIV-1 RNA molecule expression in U1 cells.** A** Basal expression of HIV-1 RNA molecules in untreated U1 (NT, LTR1, LTR4, LTR5) cells. **B** HIV-1 RNA molecules in U1 (NT, LTR1, LTR4, LTR5) cells reactivated with 1 µM of IngB 48 h. The statistical analysis was performed using one-way ANOVA comparing mean of NT with LTR1 to LTR5 (*p < 0.05; **p < 0.003***, p < 0.0003). The mean values of two technical measures of three independent biological experiments are shown

To examine whether the reactivation repression visualized in the J.Lat cell model could be replicated in a myeloid lineage, we transduced U1 cells with the LTR constructs that performed better in J.Lat10.6 (LTR1, LTR4, LTR5), stimulated with IngB and measured reactivation by accessing HIV-1 RNA expression by qRT PCR compared to untreated controls (Fig. 4). In a non-reactivated state, U1 NT cells express 4 × 10^5^ HIV-1 RNA copies/mL, while in LTR1 and LTR4 transduced cells, expression was 1.6 × 10^5^ and 1.9 × 10^5^ RNA copies/mL, respectively, reflecting an approximate two-fold reduction in HIV-1 RNA molecules expression when compared with U1 NT cells. Additionally, even in non-reactivated state, we observed that in the LTR5 transduced cells, the expression of HIV-1 RNA molecules was reduced by more than two-fold compared with U1 NT cells in all three replicates (p < 0.09). When we reactivated cells with IngB, we observed a reduction of 100-fold in HIV-1 RNA molecules expression in U1 LTR5 cells—1.5 × 10^6^ RNA molecules/mL comparing to U1 NT cells IngB treated, 1.5 × 10^8^ molecules/mL (*p* < 0.0036). There was no significant alteration in the HIV-1 RNA molecules production in IngB treated U1 LTR1 and LTR4 cells in comparison of U1 NT cells.

## Discussion

Here, we describe a potential use of the KRAB domain in association of CRISPR/dSpCas9 DNA recognition system as an attempt of a “block and lock” strategy to achieve a HIV functional cure. This system was firstly used for controlling a pseudo-type and a wild-type NL4-3 reporter HIV-1 virus in a HEK 293 T CD4^+^/CCR5^+^ cells [26]. Otherwise, our approach used lymphocytes and monocytes lineages chronically infected with a HIV-1 R7 reporter and wild-type HXB2 virus, respectively, in a latency state. J.Lat is the most used lymphocyte lineage to test LRA responses since this cell line carries a latent integrated HIV-1 with a GFP reporter instead of the Env gene. As previously reported different types of J.Lat cells show different reactivation patterns, being J.Lat 10.6 the most responsive clone, displaying a robust GFP expression after phorbol esters (PMA and IngB) stimulation (on average 80–90% activation) [22, 25, 27, 28]. Rather than induce reactivation, our aim was to develop tools for a “block and lock” approach, preventing HIV-1 RNA reactivation even in the presence of an optimal concentration of LRAs. One of our five designed CRISPR constructs (Table 1) for the HIV-1 subtype B genome could inhibit latency reactivation process 160 times (Fig. 1C, D) under PMA and IngB stimuli. Additionally, the best sgRNA interacts exactly in the LTR enhancer region, specifically in the NFκB ligation sequence and is located − 92 bp downstream of HIV-1 TSS, which could explain why the repression rate was so robust when J.Lat 10.6 LTR5 cells were treated with PMA and IngB, both NFκB stimulation drugs. This finding is consistent with the results obtained by Saayman et al. which showed that the most responsive sgRNA was the one designed for the NFκB ligation LTR’s sequence [29]. Another paper that confirms why our LTR5 sgRNA showed the greater repression effect was published by Gilbert et al. which concluded that dCas9 KRAB repressor showed robust repression ranging − 200 to  + 100 bp downstream of TSS [19].

It is well known that not only PKC agonists promotes HIV-1 reactivation, hence we analysed whether the HDACi could be capable to stimulate latent-integrated HIV-1 reactivation in J.lat 10.6 cells transduced with the LTR5 construct [30, 31]. When we added SAHA and Panobinostat, both HDACi, in LTR5 JLat cells, we observed only 1% and 3% of GFP expressing cells, respectively, showing a robust repression in this HDACi system as well we noted in PMA and Ingenol reactivation.

Despite the controversy around myeloid cells acting as latency reservoirs, some research showed that in Gut Associated Lymphoid Tissue (GALT), lung, adipose tissue, and central nervous system (CNS), these cells could promote viral latency. Myeloid cells as a latency sanctuary was also demonstrated in SIVmac infections in vivo [32]. Our work also accessed the sgRNA/dSpCas9 KRAB system in a myeloid lineage. The results obtained with U1 transduced cells, show that LTR5 sgRNA/dSpCas9 KRAB can attenuate phorbol-ester induced HIV-1 reactivation. Although we did not see the same range of repression seen using J.Lat 10.6 cells, we observed that LTR5 sgRNA can function similarly in both lineages (Fig. 4B). The difference between the repressive state in J.Lat and in U1 cells could be explained by the distinct molecular mechanisms used in latency establishment between these cell lineages [33, 34]. In terms of U1 untreated cells, we believe we were unable to observe a statistically significant reduction (p < 0.09) of HIV-1 RNA molecules in U1 LTR5 clones due to one outlier result, so this needs to be readdressed in the future (Fig. 4A).

The impact of KRAB domain in the HIV-1 latency establishment has been demonstrated by several studies. Genome-intact provirus reservoirs of 64 HIV-1 elite controllers were often integrated in centromeric satellite DNA, in genes encoding for KRAB-ZNF proteins or in heterochromatin locations rich in repressive histone markers H3K9me3 and H3K4me1 [35]. Interestingly, both repressive markers found in elite controllers are also induced by KRAB domain that are encoded in our repressive vector. Moreover, KRAB-containing zinc finger protein ZNF304 was found as a naturally robust HIV-1 latency inducer in a genome-wide CRISPR knockout screening performed using HIV-1 infected Jurkat cell line [36]. Another KRAB-containing zinc finger protein, ZNF10, was shown to repress LTR activity through interaction with NF-kB and SP1 binding sequences [37]. We believe that KAP1- a protein recruited by KRAB, and reported to be responsible by the impairment of HIV-1 gene transcription by inhibiting Tat’s P-TEFb induction- functions in both myeloid and lymphoid cells [38, 39]. In addition, in myeloid cells, KAP1, in association with CTIP2, stimulates Tat degradation [38, 40]. Recent findings, obtained with prototype foamy virus (PFV) complement our results, as they showed that a CRISPR/dCas9 fused KRAB domain, protein negatively regulates PFV transactivator protein (Tas) leading its to degradation through ubiquitination [41]. In agreement with previous studies which prove that KRAB domain is essential for HIV-1 latency, our study demonstrated that our KRAB fused CRISPR constructs displayed a robust HIV-1 transcription impairment, especially when targeted to the NFkB ligation region. Furthermore, when KRAB domain was removed from the system, the impact of dCas9 on HIV-1 reactivation was less relevant for all the LTR recognition CRISPR clones, although in J.Lat 10.6 LTR5 ko/KRAB cells we observed a significant decrease in HIV-1 reactivation compared with NT clone, but in comparison with LTR5 the presence of KRAB domain was responsible for the strong repression (Fig. 2B, C). The effect observed in LTR5 ko/KRAB cell could be explained by previous studies that demonstrated the steric inhibition of transcription promoted by dCas9 [42, 43].

Our results are also consistent with those observed by Olson et al. which suggest a relevant inhibition of KRAB-fused CRISPR/dCas9 system in J.Lat 6.3 model, when reactivated with PMA plus ionomycin. Moreover, this study showed the promotion of H3K9me3 modification mediated by their constructs in HEK293T cells [44]. Our work went a step further, showing the KRAB-fused CRISPR/dCas9 mediated repression in a robust activation responsive J.Lat 10.6 model, and in an infection competent HIV-1 myeloid latency model, U1.

The present study does not access all epigenetics patterns stimulated by the ligation of KRAB fused CRISPR/dCas9 systems on HIV-1 LTR. However, we hypothesize that KRAB-KAP1 interaction plays a critical role in the reactivation of the repressed state since removing the KRAB domain from the system diminished the repression observed upon LRA stimulus.

Further experiments such as a chromatin immunoprecipitation assay associated with next generation sequencing could be done to access the LTR5 sgRNA ligation specificity and eliminate off-target association. Design new sgRNA near HIV-1 TSS could improve the cellular repression state. An ex vivo approach using CD4^+^T cells and macrophage cells from HIV-1 infected patients could also be used to test LTR5 sgRNA construct in order to see if the results could be replicated in vivo.

Despite the fact that it was not the aim of this paper, it is worth to mention that CRISPR/dSpCas9 fused to KRAB domain system is a viable tool to future implementation in clinical trials, however some major limitation must be overcome, the necessity of constitutively expression of repressor system and the possibility off target ligation.

## Conclusion

In summary, we demonstrate a robust impact of KRAB-fused CRISPRdCas9 system in HIV-1 latency maintenance in both lymphoid and myeloid HIV-1 latency models. Additionally, we show that this system resists both types of HIV-1 LRA such as PKC agonists and HDAC inhibitors. Altogether, our constructs and results contribute with a further understanding of how HIV reservoirs would behave in a “block and lock” strategy, providing a further step towards the direction of a cure for HIV-1 infection.

## Supplementary Information

**Additional file 1: **sgRNA oligos and primers used for sequencing and PCR proceedings.

**Additional file 2: **98 design sgRNA from CRISPRPick tool.

**Additional file 3: Figure S1.** PCR confirmation of sgRNA vector transduction. 2% of agorese gel stainning with Gel Red Safer Dye. Molecular Weight (MW)—Bench Top 100 bp DNA Ladder. 1—J.Lat10.6 NT, 2—J.Lat 10.6 LTR1, 3—J.Lat 10.6 LTR2, 4—J.Lat 10.6 LTR4, 5—J.Lat 10.6 LTR5, 6—J.Lat 10.6 AAVS1, 7—J.Lat 10.6 LTR2 Ko/KRAB, 8—J.Lat 10.6 LTR3 Ko/KRAB, 9—J.Lat 10.6 LTR4 Ko/KRAB, 10—J.Lat 10.6 LTR5 Ko/KRAB, 11—U1 NT, 12—U1 LTR1, 13—U1 LTR2, 14—U1 LTR4, 15—U1 LTR5.

## Data Availability

All data generated or analyzed during this study are included in this published article and its additional information files.

## Abbreviations

KRAB: Krüppel associated box
KAP1: KRAB associated protein 1
CRISPR: Clustered regularly interspaced short palindromic repeats
PMA: Phorbol 12-Myristate 13-Acetate
HDAC: Histone deacetylase
LRA: Latency reactivation agents
DNMT: DNA methyltransferase
HMT: Histone methyltransferase
LTR: Long terminal repeat
h: Hours
min: Minutes
s: Seconds
TSS: Transcription star site
