# Supplementary figures and images for: Repression of HIV-1 reactivation mediated by CRISPR/dCas9-KRAB in lymphoid and myeloid cell models
Source: Retrovirology. 2022 Jun 22;19:12. doi: 10.1186/s12977-022-00600-9 (PMC9215058; doi:10.1186/s12977-022-00600-9)

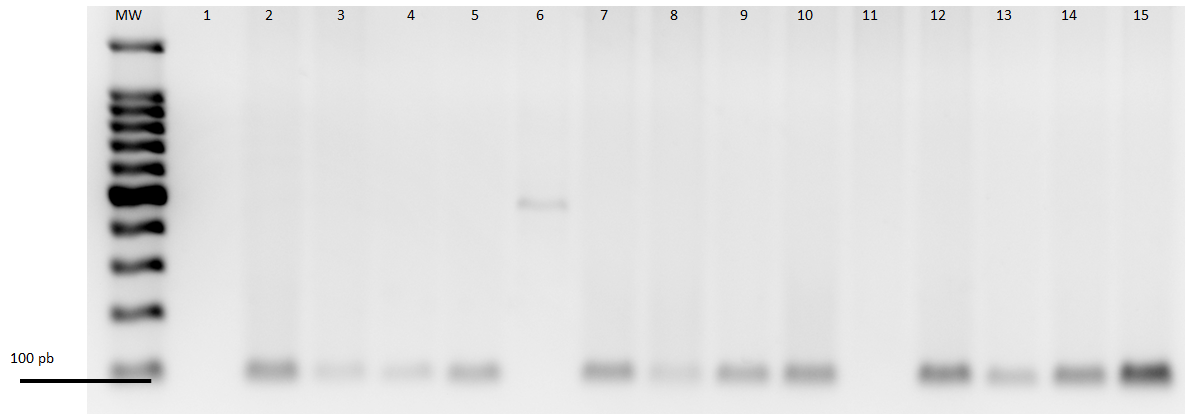

Supplement: Supplementary file 3 — Additional file 3: Figure S1. PCR confirmation of sgRNA vector transduction. 2% of agorese gel stainning with Gel Red Safer Dye. Molecular Weight (MW)—Bench Top 100 bp DNA Ladder. 1—J.Lat10.6 NT, 2—J.Lat 10.6 LTR1, 3—J.Lat 10.6 LTR2, 4—J.Lat 10.6 LTR4, 5—J.Lat 10.6 LTR5, 6—J.Lat 10.6 AAVS1, 7—J.Lat 10.6 LTR2 Ko/KRAB, 8—J.Lat 10.6 LTR3 Ko/KRAB, 9—J.Lat 10.6 LTR4 Ko/KRAB, 10—J.Lat 10.6 LTR5 Ko/KRAB, 11—U1 NT, 12—U1 LTR1, 13—U1 LTR2, 14—U1 LTR4, 15—U1 LTR5. [file 12977_2022_600_MOESM3_ESM.tif]
